# A real-world intention-to-treat analysis of a decade’s experience of treatment of hepatitis C with interferon-based therapies

**DOI:** 10.12688/f1000research.9114.1

**Published:** 2016-08-24

**Authors:** Nowlan Selvapatt, Ashley Brown

**Affiliations:** 1 Liver and Antiviral Unit, Imperial College NHS Trust Liver Unit, St Mary’s Hospital, London, W2 1NY, UK; 2 Department of Hepatology, Imperial College, St Mary’s Hospital, London, W2 1NY, UK

**Keywords:** hepatitis C virus, interferon, ribavirin, real world

## Abstract

**Objectives: **To assess the uptake of pegylated interferon (PegIFN) plus ribavirin (RBV)-based regimens in patients with hepatitis C virus (HCV) in a large, single-centre, real-world setting over 10 years.

**Methods: **This was a single centre, retrospective analysis of data from patients who attended their first appointment for treatment of HCV genotype 1–3 between 2003 and 2013. Patients were stratified by HCV genotype. The total number of patients who attended their first appointment, incidence of patients who did not proceed to treatment and associated reasons, and incidence of patients treated were analysed. Sustained virological response (SVR) rates were also reported for all patient populations.

**Results: **Overall, 1,132 patients attended their first appointment; 47.8% were included in the genotype 1 group (genotype 1a: 22.2%, genotype 1b: 13.3%, genotype 1 other: 12.3%), 7.7% in the genotype 2 group and 44.5% in the genotype 3 group. A greater proportion of patients received treatment versus those who did not receive treatment (84.4% vs 15.6%, respectively). Reasons for declining treatment included: patient declined treatment with PegIFN plus RBV: 35.0%, medical contraindications: 20.3% and mental health-related contraindications: 13.6%. An SVR was achieved in 52.6% of patients who attended their first appointment and 62.3% of patients who received treatment.

**Conclusions: **Approximately half of the patients included in this study achieved an SVR. A noteworthy proportion of patients did not receive treatment due to a reluctance to receive PegIFN plus RBV or contraindications to therapy. Results suggest an ongoing need for improvement in the treatment uptake and overall outcomes – particularly for genotype 2 and 3 patients for whom availability of interferon-free regimens is limited. The introduction of more tolerable direct-acting antiviral regimes may help overcome barriers to uptake demonstrated within this cohort.

## Introduction

Data from the World Health Organization suggest that 130–150 million people are infected with chronic hepatitis C worldwide, a significant proportion of whom will develop liver cirrhosis or cancer ^1^. Furthermore, the global burden of diseases, injuries, and risk factors study showed that in 2010 alone, an estimated 499,000 deaths were related to chronic hepatitis C ^2^. The most recent estimates from the UK suggest that 214,000 people are chronically infected with hepatitis C virus (HCV) nationally; approximately 90% are genotype 1 and genotype 3 infections ^3^.

There are seven known genotypes of HCV, although it is possible for patients to be infected with more than one genotype concurrently ^1, 4^. Treatment of HCV can be complex as the genotypes do not respond in the same way to some therapies. The armamentarium against HCV now comprises antiviral treatments that can cure approximately 90% of HCV infections, thereby reducing the risk of death from liver cancer and cirrhosis; however, global access to diagnosis and treatment remains poor ^1^.

Until 2011, the only approved treatment option for patients infected with HCV was a pegylated interferon (PegIFN) plus ribavirin (RBV)-based regimen administered for 48 weeks for genotype 1, and 24 weeks for genotypes 2 and 3. Sustained virological response (SVR) rates reported in the registration studies for the dual therapy, PegIFN plus RBV, were 42–52% for genotype 1 and 76–88% for genotypes 2 and 3 ^5– 7^. This dual therapy has been associated with frequent and sometimes serious side effects. These side effects, together with treatment durations of up to 1 year and a number of contraindications to treatment, are often seen as barriers to treatment uptake and adherence for some patients ^5– 9^.

In 2011, two first-generation protease inhibitors, telaprevir and boceprevir, were licensed for use alongside PegIFN plus RBV for patients with HCV genotype 1. This triple therapy improved SVR rates for genotype 1 patients from 42–52% to 66–75% ^5– 7, 10^; however, the tolerability profiles and contraindications for use of the first-generation triple therapies remain an issue, limiting the number of patients considered suitable for treatment ^11^. Further advances were made in the treatment options for genotype 1 patients with the introduction of IFN-free, direct-acting antiviral regimens in 2013 that have significantly improved treatment uptake, SVR rates and tolerability profiles compared with the previously available dual and triple therapies ^12– 14^. However, the availability of these IFN-free regimens is limited for treatment-naïve, genotype 2 or 3, patients in the UK.

Although patients with HCV genotype 1 now have alternative treatment options, patients diagnosed with HCV in the real world who do not qualify for treatment with new direct-acting antivirals often decline treatment with a PegIFN plus RBV-based regimen, as they are unwilling or feel unable to endure the associated side effects. Medical and mental health-related contraindications also pose a barrier to the treatment of a proportion of the HCV-infected cohort.

This study was designed to assess the uptake of PegIFN plus RBV-based regimens in patients with chronic hepatitis C in a large, single centre, real-world setting over 10 years of treatment. SVR rates for the intention-to-treat (ITT) and treated-patient populations were compared with those achieved in randomised, controlled trials using similar treatment regimens to determine whether our real-world outcomes for patients with HCV were reflective of those achieved in randomised controlled trials.

## Methods

This study was a single centre, retrospective analysis of data from patients who were referred to, and attended their first appointment at the Liver and Antiviral Unit at St Mary’s Hospital, London (part of the Imperial College Healthcare NHS Trust), for treatment of HCV genotype 1–3 between 2003 and 2013. All treatments and follow-up appointments were also carried out in the Liver and Antiviral Unit at St Mary’s Hospital. Informed patient consent was not required as no patient identifiable information was collected and data collection was retrospective for service evaluation. The work was originally commissioned as a service evaluation by the Chief of Service for Hepatology, Professor Mark Thursz, who granted permission to use and publish the data. As part of a service evaluation, ethical approval was not required. Procedures followed were in accordance with the ethical standards of clinical treatment and within the Helsinki Declaration of 1975, as revised in 2013. It is the belief of the authors that the results of this evaluation is of interest to the wider medical community.

### Patient eligibility and treatments

Referred patients ≥18 years old with virologically confirmed chronic hepatitis C genotype 1–3 were eligible for inclusion in the study. Analysis of the data for patients with genotype 4 HCV have been previously published and so were not reported in this study ^16^. The study aimed to assess all patients who were referred to the Antiviral Unit specifically for consideration of treatment by a treating hepatologist or specialist practitioner from the outpatient clinic. Therefore, all patients included in the analysis would have been seen in the outpatient setting by a specialist who intended to treat with PegIFN plus RBV. This, therefore, excluded patients who did not attend or comply with outpatient procedures or who had been deemed unsuitable for treatment by the treating physician. Patients who were referred for treatment but did not attend their first appointment at the Antiviral Unit were not included in this analysis. To ensure that the data analysed only related to patients offered an IFN-based treatment regimen (with or without first-generation protease inhibitors), patients referred for treatment after 2013 were not included in these analyses. All patients referred for treatment were screened for medical and mental health-related contraindications. Patients considered suitable for treatment were offered a PegIFN plus RBV treatment regimen over 24–48 weeks, dependent on genotype and predicted response to treatment. In 2011, when first-generation protease inhibitors became available for use in clinical practice, patients with HCV genotype 1 were offered the opportunity to include boceprevir or telaprevir in their treatment regimen.

### Study design and analyses

Data were collected on all referrals to the Liver and Antiviral Unit using information from clinical letters and prospectively collated into a computer-based database during the study period. Database and clinical note analyses were performed to establish the total number of patients referred for treatment who attended their first appointment, the incidence of patients who did not proceed to treatment and reasons thereof, and the incidence of patients treated. The incidence of patients who achieved or failed to achieve an SVR were also reported. Analyses were undertaken on the treated patients in the genotype 1 group to establish the proportion of patients whose treatment regimen included a first-generation protease inhibitor and the SVR rates thereof. Patients were considered to have achieved an SVR if they exhibited undetectable HCV RNA 24 weeks after the completion of their antiviral therapy. All analyses were descriptive and calculations were performed using Microsoft Excel 2016 software.

The reasons given for the patients who did not receive treatment were also investigated. Patient notes were used to identify medical and mental health-related contraindications; no retrospective assessments of clinical information were carried out. Therefore, contraindications were only included if they were clearly stated in the notes by the treating medical team. When a clear reason for the patient not receiving treatment was not in the notes the reason was categorised as ‘unknown’. The cirrhotic status of the untreated patients was analysed. A patient was considered to have cirrhosis of the liver in cases where the liver biopsy ISHAK score was 5 or 6 out of 6, or the pathologist reported cirrhosis, or where a Fibroscan score was >12.4 KpA.

### Patient populations

Analyses were undertaken using the ITT population, which included all patients who were referred for treatment and attended their first appointment at the Liver and Antiviral Unit. Analyses were repeated using the treated-patient population, which included patients who were referred for treatment, attended their first appointment and went on to receive treatment.

Patients were stratified by HCV genotype; patients with HCV genotype 1a or 1b were included in their respective subgroups (genotype 1a and genotype 1b). All other genotype 1 patients, including mixed genotype and other subgroups, were included in the ‘genotype 1 other’ subgroup. The genotype 1a, genotype 1b and ‘genotype 1 other’ patient populations collectively made up the overall genotype 1 group. Patients with HCV genotype 2 were included in the genotype 2 group and patients with HCV genotype 3 were included in the genotype 3 group.

## Results

**Figure 1.**
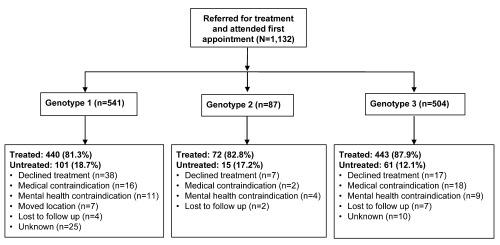
Flow and stratification of patients in the study (N=1,132). Overall, a greater proportion of patients received treatment versus those who did not receive treatment.

A total of 1,132 patients with HCV genotypes 1–3 were referred to the Liver and Antiviral Unit for treatment between 2003 and 2013. Of these patients, 47.8% were included in the genotype 1 group (genotype 1a: 22.2%, genotype 1b: 13.3%, genotype 1 other: 12.3%), 7.7% were included in the genotype 2 group and 44.5% were included in the genotype 3 group ( Figure 1, Dataset 1, Data file 1). Overall, a greater proportion of patients received treatment compared with those who did not receive treatment (84.4% vs 15.6%, respectively). A similar pattern was seen in the patient groups stratified by genotype (genotype 1: 81.3% vs 18.7%, genotype 2: 82.8% vs 17.2%, genotype 3: 87.9% vs 12.1%, respectively).

### Intention-to-treat population

**Figure 2.**
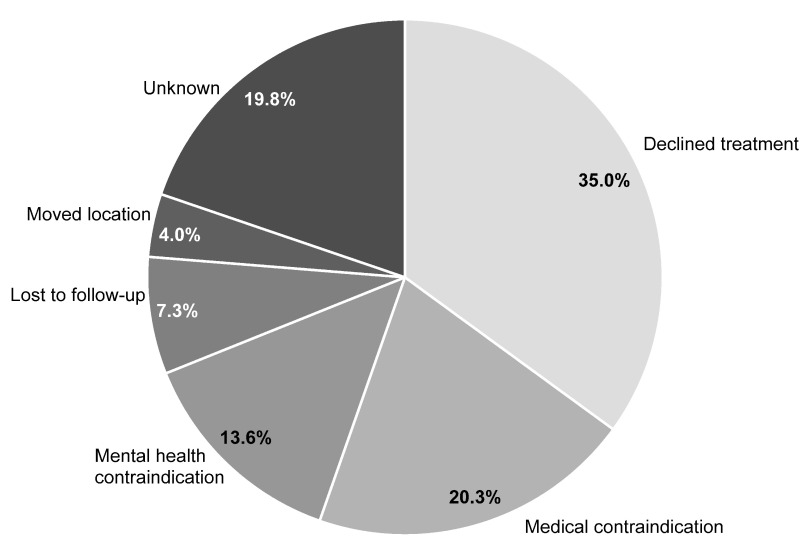
Reasons recorded for patients attending their first appointment but not proceeding to treatment (N=177). The most frequently cited reasons for not proceeding to treatment were: the patient declined treatment with PegIFN plus RBV, medical contraindication, and mental health-related contraindication. PegIFN: pegylated interferon, RBV: ribavirin.

Of the 1,132 patients who were referred for treatment and attended their first appointment, 15.6% did not receive treatment. The most frequently cited reasons were patient declined treatment with PegIFN plus RBV (35.0%), medical contraindication (20.3%) and mental health-related contraindication (13.6%) ( Figure 2, Dataset 1, Data file 2). These most frequently cited reasons for patients not receiving treatment remained consistent across the groups when stratified by genotype ( Figure 1).

Of the patients who did not receive treatment, 17.5% had cirrhosis, 42.9% did not have cirrhosis and 39.6% did not have a cirrhotic status indicated in their notes. In the groups stratified by genotype, cirrhosis was indicated in 18.8% of the patients in the genotype 1 group, no patients in the genotype 2 group and 19.7% of the patients in the genotype 3 group. This was compared with 49.5% of patients in the genotype 1 group, 60.0% of patients in the genotype 2 group and 27.9% of patients in the genotype 3 group who did not have cirrhosis. In the genotype 1 group, 31.7% had no cirrhotic status indicated in their notes, compared with 40.0% of patients in the genotype 2 group and 52.4% of patients in the genotype 3 group ( Dataset 1, Data file 3).

**Figure 3.**
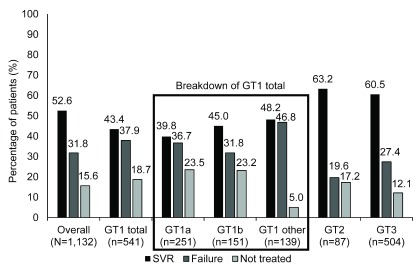
Sustained virological response and treatment failure rates stratified by genotype for the intention-to-treat population (N=1,132). The proportion of patients who achieved an SVR was higher in the genotype 2 and genotype 3 groups than in the genotype 1 group. Patients were considered to have achieved an SVR if they exhibited undetectable HCV-RNA 24 weeks after completion of antiviral therapy. The ‘GT 1 other’ group included all genotype 1 patients including mixed genotypes and other subgroups that were not genotype 1a or 1b. GT: genotype, HCV-RNA: hepatitis C virus-ribonucleic acid, SVR: sustained virological response.

In this real-world study, an SVR was achieved in 52.6% of the patients who were referred for treatment and attended their first appointment. The proportion of patients who achieved an SVR was higher in the genotype 2 and genotype 3 groups compared with the genotype 1 group (63.2% and 60.5% vs 43.4%) ( Figure 3, Dataset 1, Data file 4).

### Treated-patient population

**Figure 4.**
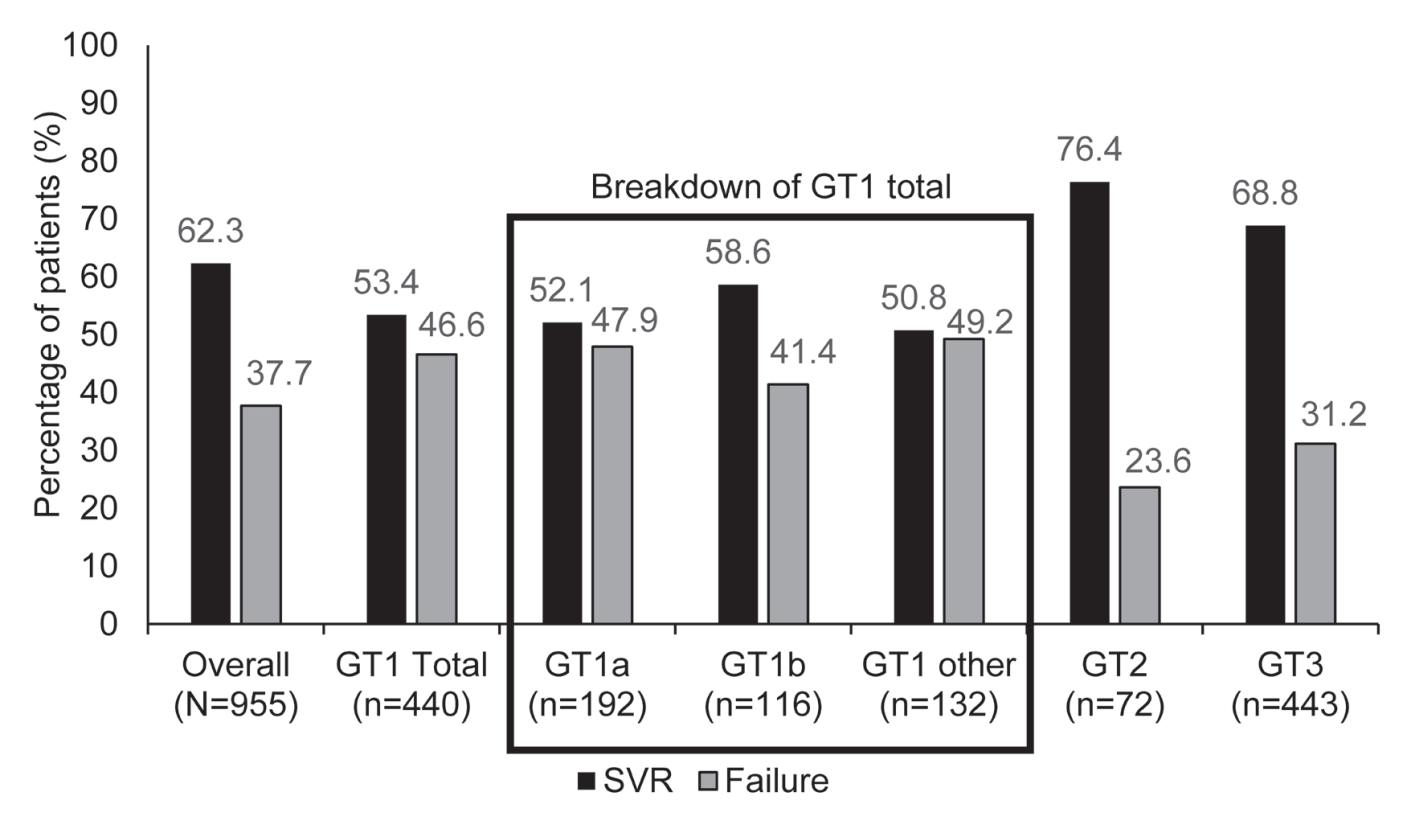
Sustained virological response and treatment failure rates stratified by genotype for the treated-patient population (N=955). The proportion of patients who achieved an SVR was higher in the genotype 2 and genotype 3 groups than in the genotype 1 group. Patients were considered to have achieved an SVR if they exhibited undetectable HCV-RNA 24 weeks after completion of antiviral therapy. The ‘GT 1 other’ group included all genotype 1 patients including mixed genotypes and other subgroups that were not genotype 1a or 1b. GT: genotype, HCV-RNA: hepatitis C virus-ribonucleic acid, SVR: sustained virological response.

Overall, 955 patients in this study received treatment and were included in the treated-patient population. Of these patients who received treatment 62.3% achieved an SVR. The proportion of patients achieving an SVR in the groups stratified by genotype was higher in the genotype 2 and genotype 3 groups compared with the genotype 1 group (76.4% and 68.8% vs 53.4%, respectively) ( Figure 4, Dataset 1, Data file 5).

**Figure 5.**
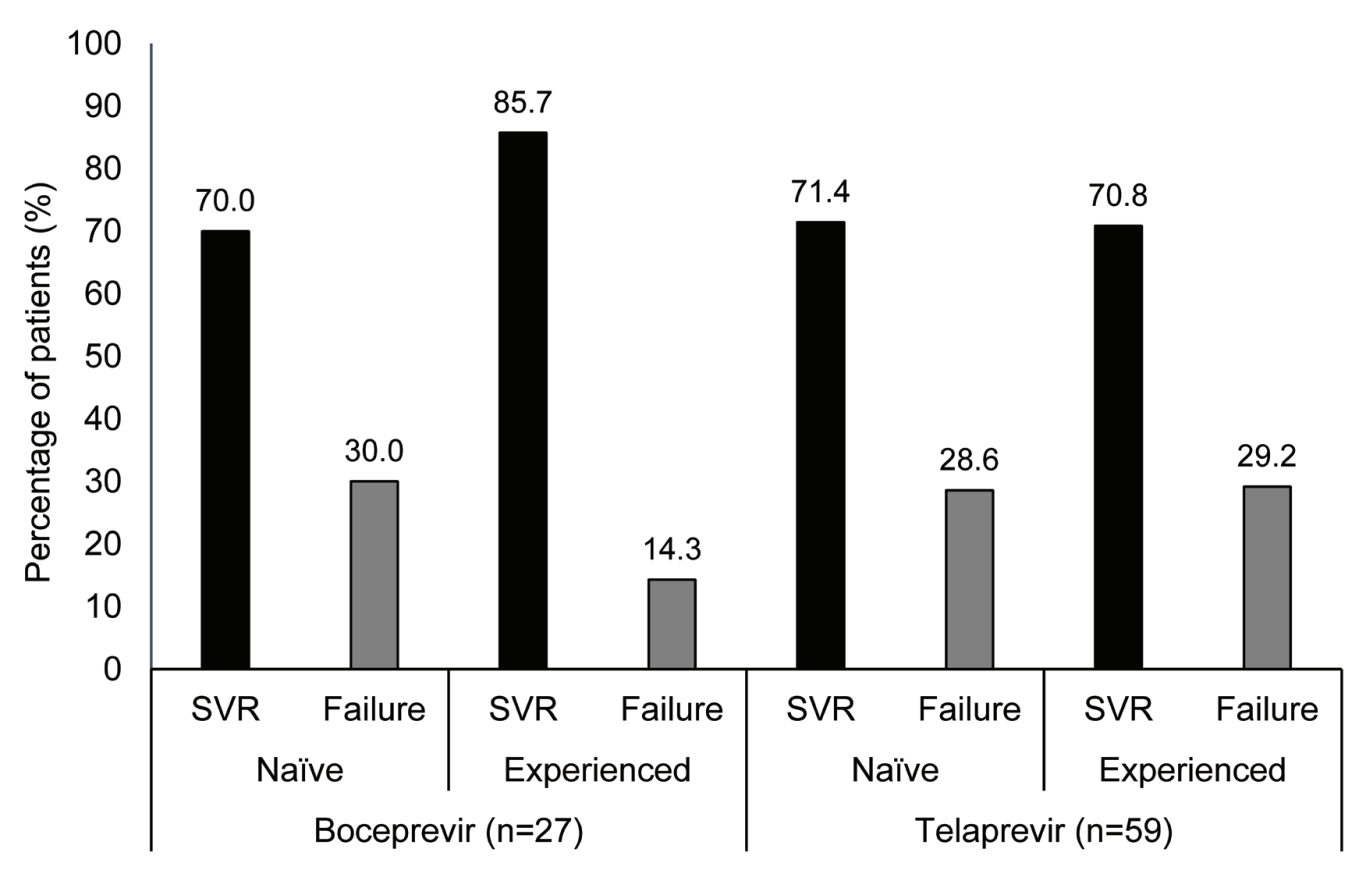
Sustained virological response and treatment-failure rates for genotype 1 patients receiving protease inhibitors (n=86). An SVR was achieved in approximately three-quarters of genotype 1 patients receiving protease inhibitors. Similar SVR rates were achieved with the regimens including boceprevir or telaprevir. Patients were considered to have achieved an SVR if they exhibited undetectable HCV-RNA 24 weeks after completion of antiviral therapy. HCV-RNA: hepatitis C virus-ribonucleic acid, SVR: sustained virological response.

Protease inhibitors were administered to 19.5% (n=86) of the treated patients in the genotype 1 group. Boceprevir was administered to 6.1% of the treated patients in the genotype 1 group and telaprevir was administered to 13.4% of the treated patients in the genotype 1 group. Overall, 72.1% of the patients who received one of these first-generation protease inhibitors achieved an SVR. Similar SVR rates were achieved with the regimens including boceprevir compared with telaprevir (74.1% vs 71.2%, respectively). Further results are presented in Figure 5 ( Dataset 1, Data file 6).

Data for real world intention-to-treat analysis of a decade's experience of treatment of hepatitis C with interferon-based therapies Underlying source data are provided. All data were collated in a computer-based database. Analyses were descriptive and calculations were performed using Microsoft Excel 2016 software. Description of each file can be found in the 'data description' file provided. Click here for additional data file. Copyright: © 2016 Selvapatt N and Brown A 2016 Data associated with the article are available under the terms of the Creative Commons Zero "No rights reserved" data waiver (CC0 1.0 Public domain dedication).

## Discussion

Results of this real-world, single centre, retrospective analysis of data from a 10-year period show that approximately 85% of patients who attended the Liver and Antiviral Unit for treatment of HCV received treatment. Data from Public Health England’s commissioning template for estimating disease prevalence suggest that the catchment area for the study centre (North West London boroughs of Barnet, Brent and Harrow) has an estimated 5,035 hepatitis C-infected individuals (n=1,602, n=208; n=1,504, genotypes 1–3, respectively) ^17^. This suggests a failure to treat a large proportion of the HCV-infected population in this region when considering total treatment numbers in this centre of 955 genotype 1–3 patients from 2003–2013 and 118 genotype 4 patients from 2002–2014 ^16^. Whilst this highlights issues regarding screening and patient identification, it could also reflect sub-optimal treatment uptake rates related to tolerability issues surrounding IFN-based therapies.

Although an SVR was achieved in 62% of the treated patients, only approximately half of the patients who were referred for treatment and attended their first appointment achieved an SVR. When stratified by genotype, as expected, the proportion of patients achieving an SVR was higher in the genotype 2 and genotype 3 groups compared with the genotype 1 group.

The proportion of the treated-patient population who achieved an SVR in this study was generally in line with previously reported outcomes in clinical trials ^5– 7^. It has been suggested previously that outcomes published for treatment of HCV in clinical studies are often not reflected in real-world clinical practice ^9^. However, this centre is a Central London teaching hospital and regional hepatology referral centre with specialist antiviral clinics and dedicated clinical nurse specialists, consultants, pharmacists and a psychiatry liaison. Therefore, it is possible that the screening and support provided at this centre enabled similar outcomes for the treated-patient population to those seen in a clinical trial-based environment. This level of resource might not be available in other centres. In this study, when the patients who attended the centre for treatment of HCV but did not receive treatment were taken into account, the SVR rates were reduced by approximately 10% across all genotypes. Furthermore, the patients who were referred for treatment and did not attend their first appointment and those with HCV who were not referred for treatment at the Liver and Antiviral Unit were not included in these analyses. The inclusion of these patients would have decreased the proportion of patients achieving an SVR further. We therefore conclude that a sub-optimal number of patients diagnosed with HCV in the UK are currently achieving appropriate treatment outcomes. These findings are in line with previously published findings by other UK-based practitioners ^9^.

More recently the introduction of direct-acting antivirals have revolutionised chronic hepatitis C treatment with superior outcomes in genotypes 1–3 compared with IFN-based therapies ^18– 25^. The key paradigm shift, however, relates to the greater tolerability and acceptability of these treatments compared with IFN-based therapies ^5– 7, 9, 18– 25^. In general these drugs have a narrower side effect profile, are not affected by concomitant opiate substitution and street drug use, and have fewer contraindications ^13, 14^. Taking into consideration the improved tolerability profiles and reduced medical and mental health-related contraindications with these new treatment options, we speculate that in the future a higher proportion of patients who attend the centre for treatment of chronic hepatitis C will proceed to treatment. This will, in turn, increase the overall rate of SVR when considering both a per-protocol and an intention-to-treat perspective. In addition, although a proportion of patients who receive treatment with a PegIFN plus RBV-based regimen after a relapse achieve an SVR ^26^, it has been suggested that deferring treatment until new options are available for these patients might be preferential ^27^.

In line with national estimates published by Public Health England, over 90% of the patients in this study had HCV genotype 1 or 3 infections ^3^. The proportion of patients who received treatment was higher in the genotype 2 and 3 groups compared with the genotype 1 group. This could be due to known higher SVR rates and shorter treatment durations for patients with HCV genotype 2 and 3 compared with HCV genotype 1 ^6, 7^. Pre-2011, patients in the genotype 1 group were offered PegIFN plus RBV for 48 weeks, with a lower probability of achieving an SVR than the genotype 2 and genotype 3 groups. Towards the end of the study period, post-2011, patients in the genotype 1 group were also offered a first-generation protease inhibitor, which improved SVR rates to approximately 70%. One-fifth of the treated patients in the genotype 1 group received first-generation protease inhibitors, which raised the mean SVR rate of the patients in the genotype 1 group slightly. Approximately half of the treated patients in the genotype 1 group and three-quarters of the treated patients in the genotype 2 group achieved an SVR in this study, in line with the PegIFN plus RBV registration trials ^5– 7, 28^. In this study, the proportion of patients in the genotype 3 group who achieved an SVR was slightly lower compared with the rates reported in the registration trials (69% vs 76–88%, respectively), but were in line with other European studies reporting outcomes of patients with HCV treated with a PegIFN plus RBV-based regimen ^5– 7, 9, 28, 29^. This could be reflective of the real-world baseline demographics of this study population compared with Phase 3 clinical trial cohorts, for characteristics such as co-morbidities, age, fibrosis status, metabolic and IL-28B status.

The most commonly recorded reason for patients not receiving treatment (for over a third of patients) was a reluctance to receive a PegIFN plus RBV-based treatment regimen. This is in line with previous studies, reporting that the side effects of PegIFN plus RBV-based regimens are commonly cited as a barrier to initiation or adherence to treatment of HCV ^28, 30^. Results from a survey of treating physicians in 2010 showed that patient-related barriers, including fear of side effects, concerns regarding treatment duration and concerns regarding treatment effectiveness, were considered the most significant barrier to treatment of HCV in Western Europe ^31^. The side effects associated with PegIFN include autoimmune syndromes, neutropenia, flu-like symptoms and neuropsychiatric disorders; while RBV has been found to induce anaemia ^28, 30^. A recent meta-analysis, including results from nine, Phase 3, clinical trials of sofosbuvir-based regimens, found that the removal of PegIFN and RBV from the treatment regimen led to a substantial improvement in patient-reported health-related quality of life during treatment ^32^. This is in contrast with the substantial decrement in health-related quality of life and productivity reported for patients receiving a PegIFN plus RBV-based regimen ^32– 36^. Medical and mental health-related contraindications made up a further third of the reasons cited for patients not receiving treatment in this study. A US study of 45,690 HCV-infected patients reported that bipolar disorder, anaemia, pregnancy and neutropenia were the most frequently cited contraindications to PegIFN plus RBV-based therapy ^37^. A small proportion of patients were lost to follow-up or moved location and the remaining fifth of patients who did not receive treatment had no clear reason recorded in their patient notes. An economic model from the USA analysing work productivity of patients with HCV genotype 1 compared patients treated with an all oral direct-acting antiviral (ledipasvir/sofosbuvir)-based regimen versus no treatment. Patients with untreated HCV were reported to impose a substantial societal burden due to reduced work productivity; the model predicted that the treatment of patients using a (ledipasvir/sofosbuvir)-based regimen would result in significant cost savings from a societal perspective ^38^.

Approximately a fifth of patients with HCV genotypes 1 or 3 who did not receive treatment had cirrhosis. A recent study of HCV-infected patients showed that patients with cirrhosis who achieved an SVR had a 5-year mortality rate of 5%, rising to over 15% for patients who did not achieve an SVR. After adjustment for potential confounding factors, achieving an SVR was found to be associated with an approximately 74% decreased risk of all-cause mortality in the cirrhotic cohort ^39^. This indicates a significantly reduced risk of death for HCV-infected patients with cirrhosis who achieve an SVR, although it is important to recognise that patients with cirrhosis have been reported to show a reduced response to antiviral therapy compared to those without cirrhosis ^40^. The recent advent of all oral direct-acting antiviral treatments has increased the capability to treat patients with cirrhosis compared to IFN-based regimens, although it is not clear yet how this may alter the natural history of disease ^41, 42^.

A number of limitations should be considered for this study including the retrospective nature of the analyses and the lack of comprehensive baseline demographics. The overall treatment population included patients who attended their first appointment at the Liver and Antiviral Unit; therefore, patients who were considered eligible and referred for treatment but did not attend their first appointment, for whatever reason, were not included in these analyses. This study is likely to have significantly under-reported the number of patients overall who were not included in the intention-to-treat analysis. Given that the analyses included only those patients who were referred directly for treatment, it is likely to exclude a multitude of patients who were either not referred or self-elected not to embark on antiviral therapies before an opportunity to be referred for treatment was offered. The concern is that this group of patients may have since developed complications of the virus or remain unlinked to care and have ongoing potential to develop HCV-related complications. More tolerable and acceptable treatments, initiated at an earlier stage with less need for specialist involvement, would conceivably increase treatment uptake rates and as a result, reduce long-term disease burden. The treatment options available for patients with HCV genotype 1 changed during the study period, with the introduction of first-generation protease inhibitors in 2011; the genotype 1 data should be interpreted with this in mind.

Results from this 10-year retrospective analysis of real-world data suggest that half of the patient population who attended the Liver and Antiviral Unit for IFN-based treatment of HCV went on to achieve an SVR. Furthermore, over half of the patients with HCV genotype 1 and one-third of the patients with HCV genotypes 2 and 3 failed to achieve an SVR. A noteworthy proportion of patients did not receive treatment due to a reluctance to receive a PegIFN plus RBV-based regimen or contraindications to therapy which may not be relevant to current direct-acting antiviral treatments. Whilst interferon therapies offer reasonable treatment outcomes for carefully selected patients at a population level, issues pertaining to patient perceptions and contraindications are a barrier for upscaling of treatments. Despite these major advances in the therapeutic options available for treatment of HCV, there remains an ongoing need for improvement in the treatment uptake and overall outcomes for the HCV-infected genotype 2 and 3 patients in our UK-based centre.

## Data availability

The data referenced by this article are under copyright with the following copyright statement: Copyright: © 2016 Selvapatt N and Brown A

Data associated with the article are available under the terms of the Creative Commons Zero "No rights reserved" data waiver (CC0 1.0 Public domain dedication). 

F1000Research: Dataset 1. Data for real world intention-to-treat analysis of a decade's experience of treatment of hepatitis C with interferon-based therapies, 10.5256/f1000research.9114.d133559 ^43^
