# Supplementary material for: A real-world intention-to-treat analysis of a decade’s experience of treatment of hepatitis C with interferon-based therapies
Source: F1000Res. 2016 Aug 24;5:2061. [Version 1] doi: 10.12688/f1000research.9114.1 (PMC5034792; doi:10.12688/f1000research.9114.1)
Supplement: Peer review history

# Peer review: Referee response for version 1

**DOI:** 10.5256/f1000research.9808.r16493

**Published:** 2016-09-22

**Authors:** Andrew Ustianowski

**Affiliations:** 1 Regional Infectious Disease Unit, North Manchester General Hospital, Pennine Acute Hospitals NHS Trust, Manchester, UK

Many thanks for asking me to review this article.

 Though no longer topical (as the treatments have progressed) this paper remains of interest as it provides some real-world insights, which are unlikely to be repeated, from an era of HCV care.

 I believe it is suitable for indexing, but have some minor recommendations.

 I wonder if it is overly long in its present form, with some minor repetition in introduction & discussion, and with topics discussed that are not directly relevant to the questions posed. However this will ultimately depend on the editorial flexibility and approach of the journal.

 After reading the abstract I was especially interested in the data on those that did not receive treatment (which is largely missing from the literature), but on reading the article itself it is apparent that this data is not of such utility unfortunately. This is because there are confounders in that other specialist clinicians have already effectively excluded many individuals by not referring on for treatment. There is also the issue of not considering those that did not attend. Though unavoidable issues with the design of this research, it does relate to the issue of using ITT analyses (as the denominators used are not translatable to any other settings). It also means that comments such as ‘ *a greater proportion of patients received treatment compared with those who did not receive treatment (84.4% vs15.6%, respectively)*’ do not provide any useful information (as these individuals have effectively been pre-screened as being more suitable for therapy).

 The study examined only G1-3 and excluded G4-6. It is not clear as to why this was the case and also it means that statements such as ‘ *In line with national estimates published by Public Health England, over 90% of the patients in this study had HCV genotype 1 or 3 infections*’ should be avoided as you are not comparing like with like.

 The authors comment that their SVR rates for G1 and G2 are comparable to the trial data, potentially due to the clinical and patient support infrastructure they have in place – but that infrastructure was presumably also there for the G3 patients (who had a lower than expected SVR rate) and therefore I think such conclusions may be too speculative.

 Other comments:

  

- It is more generally accepted that there are 6 distinct genotypes of HCV – not 7 as stated in paragraph 2 of the introduction.
- The graphing in figures 4 and 5 does not need both columns as the outcome for each is dichotomous (either SVR or failure) – therefore just the SVR column would be better for clarity.
- I could not link on my computer to the datasets and so could not assess them.

I have read this submission. I believe that I have an appropriate level of expertise to confirm that it is of an acceptable scientific standard.

# Peer review: Referee response for version 1

**DOI:** 10.5256/f1000research.9808.r16119

**Published:** 2016-09-06

**Authors:** Mark Wright

**Affiliations:** 1 Department of Hepatology, Wellcome Trust Clinical Research Facility, University Hospital Southampton NHS Foundation Trust, Southampton, UK

The article stands as a final word on a closed era of HCV treatment.

 I wonder if in the rapidly changing world of HCV treatments the introduction should be updated a little, especially the second to last paragraph that implies that in the real world some patients with G1 might still be treated with PEG RIBA.

 I also question the relavence of the presenting of the data separating referrals to the anti viral unit (as a sort of ITT analysis) and those actually treated (per protocol esque analysis). This is a little artificial as we don't know how many people were seen in other hepatology clinics and never referred because they were either deemed unsuitable or uninterested.

 Other than these few tweaks I approve.

I have read this submission. I believe that I have an appropriate level of expertise to confirm that it is of an acceptable scientific standard.
